# Prednisone for Acute Complex Regional Pain Syndrome: A Retrospective Cohort Study

**DOI:** 10.1155/2020/8182569

**Published:** 2020-02-25

**Authors:** Andrew Jamroz, Michael Berger, Paul Winston

**Affiliations:** ^1^University of British Columbia, Island Medical Program, Victoria, British Columbia, Canada; ^2^Division of Physical Medicine & Rehabilitation, Department of Medicine, University of British Columbia, Vancouver, British Columbia, Canada; ^3^International Collaboration on Repair Discoveries (ICORD), University of British Columbia, Vancouver, British Columbia, Canada; ^4^School of Kinesiology, Faculty of Education, University of British Columbia, Vancouver, British Columbia, Canada

## Abstract

**Objective:**

The objective of this study was to evaluate prednisone effectiveness on complex regional pain syndrome (CRPS) features in a community-based outpatient rehabilitation setting.

**Design:**

A single-centre, retrospective inception cohort design was used. Inclusion criteria were CRPS diagnosis according to the Budapest criteria, involvement of multiple joints, treatment with prednisone, and duration of symptoms less than one year. Typical prednisone treatment was 28-day taper regimen with 60 mg. Patient symptoms and signs were compared before and after treatment.

**Results:**

There were 39 patients who met inclusion criteria for analysis. Duration of symptoms before treatment was 80.8 ± 67.7 days. Following treatment, 19 (48.7%) patients reported complete pain resolution, 19 (48.7%) patients reported decreased pain permitting functional use, and 1 (2.6%) patient reported no improvement. All symptoms and signs decreased significantly following oral prednisone treatment (*p* < 0.001). Range of motion (ROM) deficits persisted in 19 (49%) patients. However, 17 of these patients reported functional ROM recovery. Degree of ROM recovery and time-to-treatment had low positive correlation (*r* = 0.354, *p* < 0.001). Range of motion (ROM) deficits persisted in 19 (49%) patients. However, 17 of these patients reported functional ROM recovery. Degree of ROM recovery and time-to-treatment had low positive correlation (

**Conclusions:**

These data support short-course prednisone treatment for acute and subacute CRPS with multijoint involvement in a community rehabilitation setting. The association between time-to-treatment and ROM recovery suggests earlier treatment may result in improved ROM outcomes.

## 1. Introduction

Complex regional pain syndrome (CRPS) is defined as a group of regionally painful conditions disproportionate to any inciting event [1]. It presents as a constellation of pain and edema, with sensory, vasomotor, sudomotor, motor, and trophic signs and symptoms. The prognosis for CRPS is variable and ranges from early recovery within one year, to progression to chronic pain and disability [2]. Due in part to a heterogeneous patient presentation and a lack of a definitive diagnostic testing, CRPS continues to be difficult to diagnose and treat [3]. In an effort to standardize patient diagnosis, the Budapest criteria were created [1] and have been shown to provide high sensitivity and specificity [4].

Treatment of CRPS continues to be challenging for clinicians due to a lack of consensus and evidence-based therapies. Corticosteroids have been previously studied as a possible pharmacologic treatment for CRPS [5–13]. Most of these studies used small sample sizes with older CRPS diagnostic criteria, with few using the Budapest criteria [9, 14]. Furthermore, there is no consensus regarding the optimal dose of corticosteroids, treatment duration, or tapering schedule to guide clinicians [7, 11, 15]. There remains limited research evaluating the effectiveness of oral prednisone diagnosed by the Budapest criteria. In Complex Regional Pain Syndrome: Practical Diagnostic and Treatment Guidelines, Harden et al. [16] noted that oral corticosteroids are the only anti-inflammatory drugs for which there is direct clinical-trial evidence in CRPS. Despite this, prednisone treatment has not become common place [17].

Of note, CRPS diagnosis by the Budapest criteria does not require specific diagnostic tests or specialized equipment [18]. Individual physicians may triage, diagnose, and treat CRPS outside of specialty pain centers. There are no standardized outcome measures, and as Harden et al. underscored, functional restoration should be the desired outcome in CRPS rather than harder to quantify individual biometrics [16]. The purpose of this study was to evaluate the effectiveness of prednisone on diminishing the Budapest criteria CRPS signs and symptoms in early disease presentations in the community setting. It was hypothesized that prednisone treatment will lead to CRPS recovery by improving pain and clinical features.

## 2. Methods

### 2.1. Study Participants

**Figure 1:**
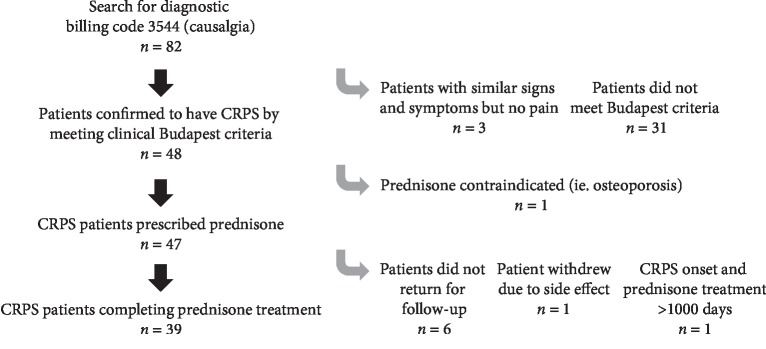
Flowchart of patients fulfilling inclusion criteria. CRPS: complex regional pain syndrome.

Participants were included in this retrospective cohort study if they fulfilled the clinical Budapest criteria (see Table 1), [1] presented with limited range of motion in more than one joint as assessed by clinical examination, were treated with prednisone upon presenting to our clinic, and returned for reassessment after completing the treatment regimen. To ensure all cases were captured from our electronic medical record, a search for the ICD code for CRPS (causalgia) was performed for all cases between May 2013 and May 2018. This study was approved by the local ethics review board. In addition, this study conforms to all STROBE [19] guidelines and reports the required information accordingly. A flow chart detailing patient enrollment is seen in Figure 1. Study data were collected and managed using Research Electronic Data Capture (REDCap) [20].

### 2.2. Treatment Regimen

The typical prednisone regimen started with 60 mg followed by taper of 5 mg per day until 20 mg. Patients then remained on 15 mg for one week, 10 mg for one week, and finally 5 mg for one week. A slightly modified decreased dose was chosen in elderly, adolescent, and diabetic patients, typically starting at 40 mg before the taper, as has been described in a previous study [10]. All patients were instructed to continue with their existing physiotherapy treatment, but we did not control for different types and frequencies of adjunct treatments. As is common practice in our centre, vitamin D and calcium were supplemented at doses of the pharmacist's discretion.

### 2.3. Outcomes

The primary outcomes were the presence or absence of Budapest criteria signs and symptoms after prednisone therapy, as compared to before prednisone treatment. CRPS symptoms were gathered from patient histories. CRPS signs were assessed by one physiatrist (PW) and extracted from the clinical records by another author (AJ). Details of sign assessment by the physiatrist can be found in Table 2. Patients were followed for the duration of prednisone therapy with additional follow-up to assess for the ongoing presence of signs and symptoms, as required.

Restoration of limb usage was the primary goal. At final follow-up, patient chart descriptions permitted stratification of pain and range of motion into three levels rather than only present or absent. Pain was stratified into “no longer present”, “decreased pain”, or “not improved”. Any residual or minimal pain was grouped as “decreased pain”, even if insignificant in the patients' day-to-day lives. Range of motion (ROM) was stratified into “fully restored”, “functionally restored”, or “not restored”. “Fully restored” ROM meant patients were able to actively demonstrate full ROM expected for healthy joints. “Functionally restored” ROM indicated patients were capable of using their affected limb for required activities but described or demonstrated residual joint stiffness. For instance, a patient could not fully tuck fingertips in their fist but were able to play tennis and use a pen. “Not restored” ROM meant the ROM limitations interfered considerably with many of the patient's activities.

### 2.4. Photographs

At each visit, deidentified photographs of the CRPS-affected limb were taken. The edema, vasomotor signs, and degree of ROM were captured in each photograph. Representative photographs were chosen to document progression of recovery.

### 2.5. Side Effects

Any adverse reactions were documented at each visit.

### 2.6. Statistics

CRPS onset was estimated as the earliest date in the patient's records when the documented signs and symptoms fulfilled the clinical Budapest criteria. Duration of follow-up was determined by calculating duration between first and last clinical encounter. To confirm reductions of CRPS signs and symptoms were statistically significant, McNemar's test on the difference of proportions, applicable to pretreatment and posttreatment, was performed. Kendall's tau *b* correlation was used to determine whether patient factors were associated with any of the outcomes at final visit. Logistic regression was used to determine if patient factors predicted outcomes at final visit. Patient factors considered were age, sex, upper/lower body, right/left side, injury mechanism (fracture vs. nonfracture), initial diagnosis (CRPS I vs CRPS II), early/late application of treatment, and short/long prednisone treatment. These factors were chosen to determine if specific patient characteristics are more responsive to prednisone treatment.

## 3. Results

Patient demographics and clinical data are provided in Table 3. In our study, 59.0% of patients completed the 60 mg prednisone taper, 33.4% completed prednisone taper starting from a lower initial dose (between 40 and 60 mg), and 7.7% of patients completed the 60 mg prednisone taper with an additional two weeks of 20 mg prednisone taper due to lingering CRPS symptoms. Of the four adolescent patients, three had CRPS confined to the lower extremity.

**Figure 2:**
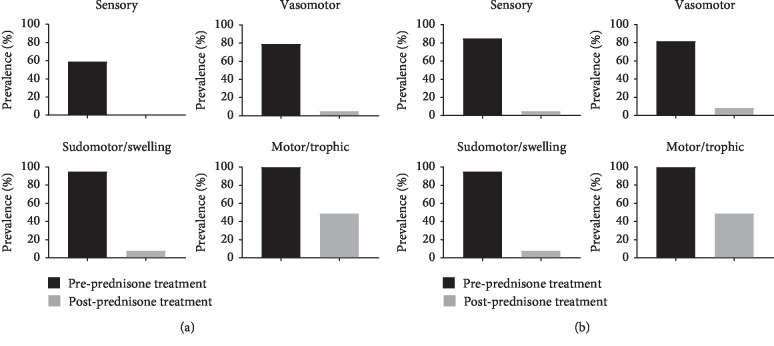
(a, b) Prevalence of complex regional pain syndrome signs and symptoms before and after short course of prednisone treatment. There is a statistically significant decrease in all signs and symptoms categories per McNemar's test (*p* < 0.001). Sensory, vasomotor, and sudomotor/edema signs and symptoms almost disappeared following prednisone therapy. (a) Signs. (b) Symptoms.

After completing prednisone treatment, only one patient (2.6%) continued to fulfil the clinical Budapest criteria. This patient had the longest duration of symptoms prior to treatment at 324 days, significantly longer than our average of 80.8 ± 67.7 days. Symptoms and signs resolved following oral prednisone treatment per McNemar's test (*p* < 0.001). The sensory, vasomotor, and sudomotor/edema signs and symptoms were present in less than 10% of patients (Figure 2). The motor/trophic signs and symptoms persisted in 19 (49%) of patients. Range of motion deficits were the specific sign and symptom that persisted in the motor/trophic category. However, 17 of these 19 patients reported functional ROM recovery (Table 4). Pain recovery following prednisone treatment is seen in Table 5. Only one patient saw no improvement in pain, whereas 48.7% of patients saw full recovery from pain.

The degree of ROM recovery and time to treatment had a low positive correlation (*r* = 0.354, *p*=0.026). No other parameter demonstrated a significant relationship with recovery (*p* > 0.05). When modeling logistic regression for each sign and symptom and pain level, none of the patient factors significantly predicted clinical outcome (*p* > 0.05).

**Figure 3:**
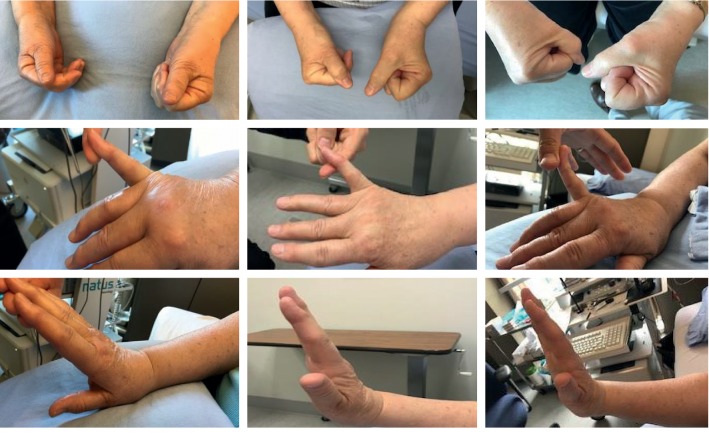
Complex regional pain syndrome type II due to carpal tunnel syndrome with multiple joint ranges affected. There is progression from before prednisone treatment (left images) to two weeks of treatment (middle images) to completion of four-week course of prednisone (right images).

**Figure 4:**
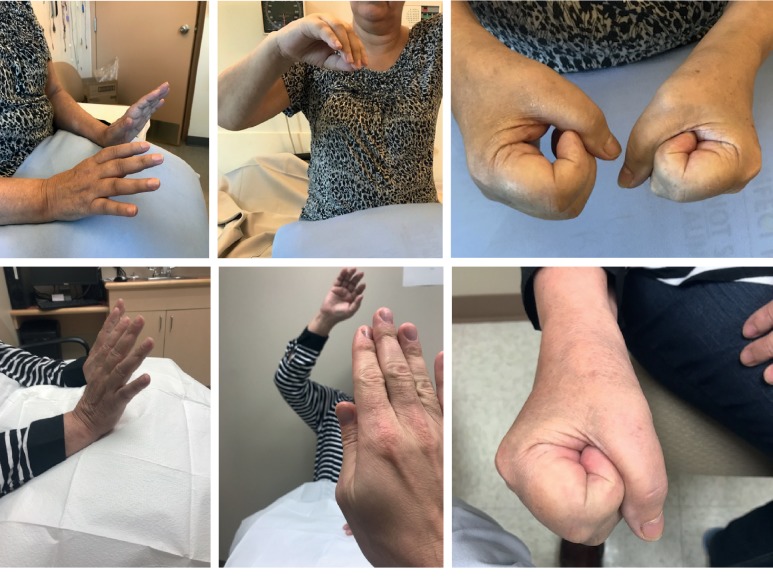
Complex regional pain syndrome after right humerus fracture with involvement of the shoulder, elbow, wrist, and fingers presenting with pain and decreased range of motion. Top images were before treatment, and bottom images were after 4-week course of prednisone.

**Figure 5:**
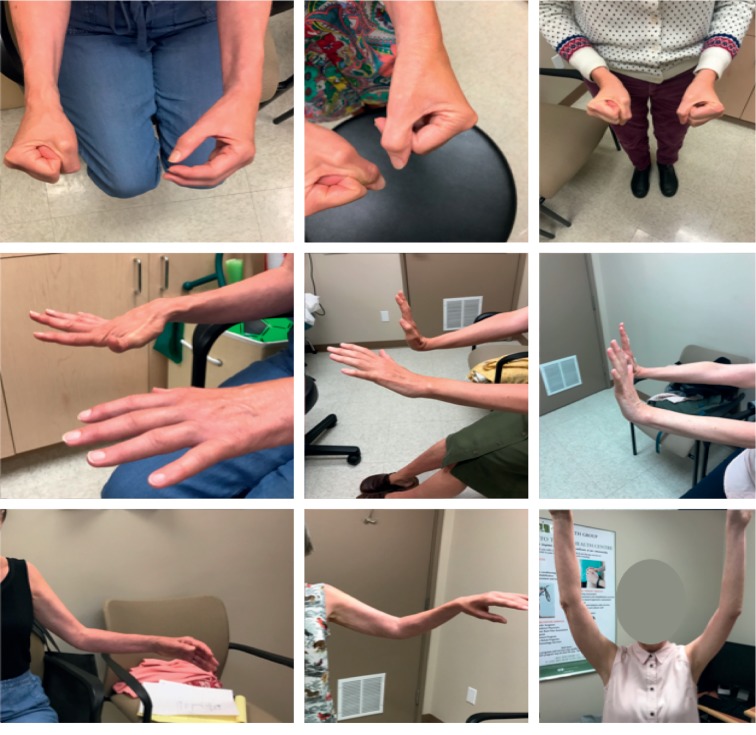
Complex regional pain syndrome after elbow dislocation. Initial presentation is shown in the first column. The patient no longer met Budapest criteria at 4 weeks on prednisone, with functionally but not fully restored range of motion (second column). However, the patient continued to recover after completion of treatment (third column).

**Figure 6:**
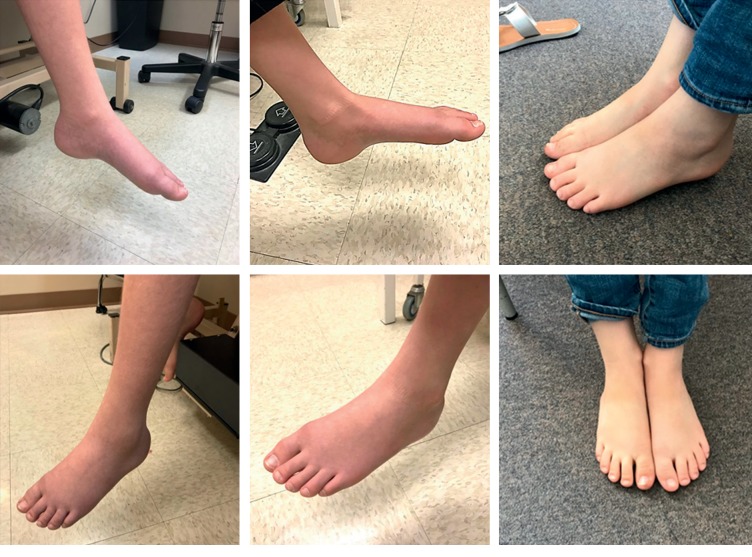
Idiopathic left leg complex regional pain syndrome in adolescent female. Left images were taken before prednisone treatment, middle images were taken at two weeks of treatment, and right images were taken at two months after starting treatment. The patient made a full recovery.

Representative photographs tracking patient response to prednisone after various inciting triggers are shown. Recovery of CRPS after carpal tunnel syndrome (Figure 3), postfracture (Figure 4), elbow dislocation (Figure 5), and idiopathic onset (Figure 6) are illustrated. Following prednisone therapy, the edema and vasomotor, motor, and trophic signs and symptoms improved. In most cases, improvement was observed at two weeks by decreased swelling and improvement in ROM. Four weeks of prednisone therapy led to CRPS resolution in most patients. Functionally restored ROM is depicted in Figure 5.

The majority of patients (71.8%) reported no side effects (Table 6).

## 4. Discussion

This study evaluated the effectiveness of prednisone for CRPS diagnosed using the current International Association for the Study of Pain (IASP) criteria (Budapest clinical criteria), in an outpatient community setting, using a retrospective cohort design. Following prednisone treatment, all but one patient no longer met these criteria (97.4%). Over 90% of patients reported functional improvement in range of motion. We found significant decreases following treatment in the CRPS clinical features. No factors such as age, sex, upper/lower body, injury mechanism, or short/long treatment duration predicted outcomes to prednisone treatment. This study builds on the existing CRPS literature to demonstrate how the Budapest criteria can be used in the community setting to facilitate early treatment and how a longer follow-up duration can prevent relapse. We highlight how this study has a similar patient population to large CRPS studies [21–23] and builds on the findings reported by previous research. Over the course of prednisone treatment, only minor side effects were observed.

Our results are suggestive of consistent prednisone effectiveness in patients diagnosed using the Budapest criteria. We note that Bean et al. [24] prospectively investigated the extent of recovery in 59 CRPS patients receiving various common CRPS treatments, with only 19.6% having received prednisone. After treatment, 25% (15/59) of these patients continued to meet the Budapest criteria. In the present study, all patients were treated with prednisone and only 2.6% (1/39) continued to fulfil the Budapest criteria after treatment.

The pain level results (Table 5) suggest a significant prednisone treatment effect, with 48.7% of patients achieving full pain recovery and another 48.7% achieving sufficiently decreased pain to permit functional usage. Barbalinardo et al. found that pain did not decrease with prednisolone treatment in patients with longstanding CRPS diagnosed by the Budapest criteria [14]. The patient population in that study had a median CRPS duration of 15 months, compared to our patient population with median CRPS duration of 1.9 months. Our patient population is considered to have been in the acute or subacute period of CRPS [25]. Our only patient that did not see positive changes was at nearly 11 months since onset. Our results hint at better outcomes than a previous study by Savas et al., which assessed patients with a mean CRPS duration of 1.9 months treated with physiotherapy modalities, manual therapies, oral analgesics, and anti-inflammatories and found that only 10% of patients were found to be free of pain at a mean duration of 18 months after treatment [26]. Our findings suggest that early treatment of prednisone is more positive than it suggested by accepted guideline care.

Our retrospective study also focused on the clinical features of CRPS before and after treatment. The sensory, vasomotor, and sudomotor/edema categories of the Budapest criteria decreased the most. The motor/trophic group features decreased significantly though not to the same extent. This reflects the ongoing stiffness in contracted joints. Our finding aligns with a systematic review of CRPS outcomes that found that motor symptoms tend to persist, whereas sudomotor and vasomotor symptoms improve [2]. Our logistic regression did not show that any factor-predicted outcome. This is most likely due to the small sample size and the finding that almost all patients were responders to treatment.

It has been speculated that prednisone treatment may be most effective only in early CRPS. [2] Bean et al. concluded treatment improvements were greatest within six months of CRPS onset [24]. We found a low positive association between time to treatment and degree of ROM recovery. As all of our patients were in the acute to subacute phase, it is possible we could not adequately measure the importance behind time to treatment. A similar situation could explain Bianchi's study, in which the maximum CRPS duration was 204 days before treatment [10]. Another study found that patients with longstanding CRPS did not respond to prednisolone [14]. The median CRPS duration before treatment was 15 months, which is far greater than our studied population. Results from all these studies hint towards the importance of time to treatment and underscore the importance of early recognition of CRPS before entering a chronic phase.

Our community-based study reflects a patient population similar to other published CRPS populations. Fracture has been reported to incite CRPS in 42% to 46% of cases [21–23]. CRPS tends to affect females more commonly than males (71% female) [21] and upper extremities are more commonly affected (70%) [21]. In our study population, 41% of patients had fracture preceding CRPS, 66.6% of patients were women, and 74.4% of patients had upper extremity involvement. As the pathophysiological mechanisms leading to CRPS development remain unknown, we did not exclude patients with idiopathic CRPS onset in line with other publications [21].

While previous studies have investigated corticosteroids as a treatment for CRPS, our study has several key differences: updated diagnostic criteria, length of follow-up, documentation of CRPS onset to treatment duration, and photographs. A retrospective study by Atalay et al. reported positive outcomes following treatment with prednisolone on 45 patients using the previous IASP CRPS criteria [11]. These diagnostic criteria are no longer endorsed by the IASP as the criteria lacked specificity [4]. In fact, most studies investigating corticosteroids for CRPS treatment do not use the CRPS Budapest criteria endorsed by the IASP [5–8, 10, 11]. The study by Atalay et al. followed patients for only the three weeks of treatment from an initial 30 mg prednisolone dose with taper. Subsequently, Kalita et al. found 50% of poststroke CRPS patients treated with 40 mg of prednisolone for two weeks and a two-week taper deteriorated after the cessation of prednisolone and required additional treatment. We also noted a subset of our patients required retreatment.

Recovery from CRPS clinical features is demonstrated in the pre- and posttreatment photographs (Figures 3–6). We used sequential photographs to demonstrate improvement in ROM and normalization of appearance of the limbs. Diagnosing CRPS remains controversial due to unknown pathophysiology, lack of definitive laboratory findings, fluctuating signs and symptoms, and varying responses to treatment [27]. Our photographs demonstrate a similar inflammatory and restricted ROM presentation across all patients. This is consistent with the findings of Bruehl et al. who reported that acute CRPS was mainly an inflammatory process [25]. Our photographs also show a predictable response to prednisone. The uniformity observed in our study is likely due to our strict inclusion criteria. The photographs also demonstrate how quickly the prednisone effect takes place on changing the appearance within a relatively short time frame. From our practice, we noticed that the largest improvement in CRPS patients occurred rapidly within two weeks when the patient was taking the prednisone therapy (Figure 3).

There is concern when prescribing a course of steroids due to contraindications and side effects. In our study, we offered a relatively short course of prednisone therapy. The majority of the patients reported no side effects with the tapering regimen (71.8%). All side effects were temporary and subsided after prednisone cessation. Only one patient withdrew from the study due to side effects (Figure 1). Our results are in line with other studies using corticosteroids for CRPS, which reported minimal to no side effects [7, 9, 11, 14]. We note that there was no difference in outcomes for patients started at 60 mg or the reduced dosage. This is quite consistent with the wide range of doses and length of treatment in previous studies, which typically range from 30 to 60 mg, though Birklein et al. used 100 mg prednisolone taper with a 25% taper every four days [15]. Lastly, we acknowledge steroid use in adolescents may be a cause of concern. The reduced starting dose of 40 mg was chosen to minimize potential side effects while still treating the debilitating CRPS.

There are several limitations in interpreting our data. First, there was no quantitative measure of pain and ROM, though we note the Budapest criteria is a clinical tool that does not provide for a specific quantitative measure. Harden explained the challenge of seeking specific biometrics in CRPS. Our primary outcome was the resolution of the signs and symptoms and return to functional limb usage. As a retrospective study of clinical practice, there are no placebo controls in this study. Confounders in this study include different durations of therapy, different times of CRPS onset to initiation of prednisone therapy, varied concurrent therapies (such as vitamin D, calcium, and physiotherapy), medication adherence, and different number of follow-up visits. This study therefore can only be interpreted as being suggestive of beneficial effects of corticosteroids in early CRPS. A controlled prospective study is needed to address the design limitations of this study and account for the influence of any confounders. Such a trial could be better suited to describe the patient population most responsive to prednisone therapy.

## 5. Conclusions

These results suggest that in acute CRPS patients diagnosed by the Budapest criteria with multijoint involvement, prednisone may be an important therapy to promote patient recovery. Our study highlights the safe and effective use of prednisone in the routine community clinic setting without specialty services or tests.

## Figures and Tables

**Table 1:** Diagnosis of complex regional pain syndrome (CRPS) by clinical Budapest criteria.

Budapest criteria signs and symptoms (IASP [1])	
Continuing pain that is disproportionate to any inciting event	
*At least one symptom in at least three of the following categories:*	
Sensory	Hyperalgesia, allodynia, altered sensation/paresthesia
Vasomotor	Temperature asymmetry, skin colour asymmetry
Sudomotor	Edema, sweating changes
Motor/trophic	Decreased range of motion, weakness, trophic changes (hair, nail, skin)
*At least one sign at time of evaluation in at least two of the following categories:*	
Sensory	Evidence of hyperalgesia to pinprick, allodynia to light touch, and paresthesia by different sensation upon application of same pressure to CRPS-affected and non-CRPS-affected area
Vasomotor	Evidence of temperature asymmetry manually and skin colour asymmetry visually by simultaneous comparison between CRPS-affected and non-CRPS-affected area
Sudomotor	Evidence of edema by observing lack of normal wrinkling, sweating changes determined by observing sweating patterns at CRPS-affected region differing from non-CRPS-affected regions
Motor/trophic	Evidence of decreased ROM with active movement, weakness determined by decreased strength of joint compared to unaffected side, decreased or increased tendon reflexes as determined by reflex response to hammer in CRPS-affected region, trophic changes of hair or nail evaluated by comparing between CRPS-affected and non-CRPS-affected regions, skin changes by presence of shiny skin
*No other diagnosis can better explain the symptoms and signs*	

**Table 2:** Detailed methods of assessing CRPS signs by physiatrist.

*Sensory*
Hyperalgesia was evaluated by increased pain response to pinprick
Allodynia was determined by painful response to light touch
Altered sensation/paresthesia was evaluated by patient noting different sensation following application of the same force on CRPS-affected and non-CRPS-affected area, usually contralateral extremity

*Vasomotor*
Temperature asymmetry was determined by simultaneous comparison between CRPS-affected and non-CRPS-affected area by clinical observation without specialized equipment
Skin colour changes were determined by visual comparison between CRPS-affected and non-CRPS-affected area

*Edema/sudomotor*
Edema was determined by observing lack of normal wrinkles, for example, at knuckles as well as generalized swelling
Sudomotor signs (sweating changes or asymmetry) were determined by observing sweating patterns at CRPS-affected region differing from non-CRPS-affected regions

*Motor/trophic*
Evaluation of range of motion (ROM) was determined by examining the active and passive ROM of all proximal and distal joints of the affected limb
Weakness was determined by decreased strength compared to unaffected side
Decreased or increased tendon reflexes were determined by reflex response to hammer in CRPS-affected region
Increase or decrease in hair growth was evaluated by comparing between CRPS-affected and non-CRPS-affected regions and confirming with the patient that this was not due to nonnatural cause (ie., shaving only one limb)
Increase or decrease in nail growth was determined by comparing between CRPS-affected and non-CRPS-affected regions and confirming with patient this was not due to nonnatural cause (ie., cutting nails on only one side of body)
Skin changes were evaluated by the presence of shiny skin, brawny, or other observed asymmetries

**Table 3:** Patient demographics and relevant variables collected for the retrospective cohort.

Variable	Duration or proportion
Average age	51.5 ± 18.7 years [11–85 years]
Female sex	66.7%
Inciting trigger (fracture)	41.0%
Inciting trigger (idiopathic)	25.6%
Inciting trigger (surgery)	12.8%
Inciting trigger (trauma)	12.8%
CRPS in upper extremity	74.4%
CRPS right side unilateral	48.7%
CRPS left side unilateral	43.6%
CRPS bilateral	7.7%
CRPS type I	79.5%
Fulfilled Budapest research criteria	59.0%
Mean duration of prednisone therapy	27.2 ± 5.3 days
Mean duration of CRPS onset to start of prednisone treatment	80.8 ± 67.7 days
Mean duration between initial and final visit	116.4 ± 159.1 days

**Table 4:** Range of motion (ROM) levels at final visit after completion of prednisone treatment. Functional range of motor recovery allowed patients to perform most activities without trouble such as using a pen or playing tennis.

Range of motion stratification	Prevalence after prednisone treatment
Fully restored ROM	51.3% (20/39)
Functionally restored ROM	43.6% (17/39)
ROM not restored	5.1% (2/39)

**Table 5:** Pain levels at final visit after completion of prednisone treatment.

Pain stratification	Prevalence after prednisone treatment
Pain no longer present	48.7% (19/39)
Decreased pain permitting functional use	48.7% (19/39)
Pain not improved	2.6% (1/39)

**Table 6:** Breakdown of the reported side effects during the course of prednisone therapy.

Side effect	Proportion
None	71.8% (28/39)
Difficulty sleeping	12.8% (5/39)
Headache	7.7% (3/39)
Weight gain	2.6% (1/39)
Nausea	2.6% (1/39)
Vomiting	2.6% (1/39)
Elevated blood glucose	2.6% (1/39)
Elevated blood pressure	2.6% (1/39)
Osteopenia	2.6% (1/39)
Anxiety	2.6% (1/39)

## Data Availability

The patient data used to support the findings of this study are restricted by the Vancouver Island Health Authority Ethics Review Board in order to protect patient privacy. Data are available from Paul Winston (pauljwinston@gmail.com) for researchers who meet the criteria for access to confidential data.
